# A Case of Trousseau Syndrome: A Report Detailing Recurrent Ischaemic Strokes Associated With Metastatic Pancreatic Cancer and Pulmonary Embolisms Despite Treatment With Direct Oral Anticoagulation

**DOI:** 10.7759/cureus.97696

**Published:** 2025-11-24

**Authors:** Ei Thinzar Aung, Morgan W Hayden, Karen Chouhan, Ye Thurein Mon, Laura Skeldon

**Affiliations:** 1 Stroke Medicine, The Grange University Hospital, Newport, GBR; 2 Internal Medicine, The Grange University Hospital, Newport, GBR; 3 Geriatrics, The Grange University Hospital, Newport, GBR

**Keywords:** anticoagulation, direct oral anticoagulant therapy, hypercoaguable state, hypercoagulable state, pancreatic ca, pulmonary embolism (pe), recurrent ischemic stroke, stroke, trousseau’s syndrome

## Abstract

Pancreatic cancer is a malignancy strongly associated with cancer-related hypercoagulability. Venous thromboses are a well-documented complication of pancreatic cancer, but there is less awareness and focus on arterial events such as ischaemic stroke and myocardial infarction. We present the case of a patient who was diagnosed with ischaemic stroke preceding a diagnosis of an occult metastatic pancreatic malignancy and concurrent pulmonary embolism. Despite treatment with therapeutic doses of direct oral anticoagulation, the patient subsequently developed recurrent bilateral ischaemic strokes. This case highlights the challenges of managing mixed venous and arterial thromboembolic disease in patients with pancreatic cancer and illustrates the aggressive, refractory nature of Trousseau syndrome.

## Introduction

Trousseau syndrome describes a malignancy-associated hypercoagulable state that leads to recurrent venous and arterial thromboses [1]. It can manifest as migratory thrombophlebitis, venous thromboembolism (VTE), and arterial thrombotic events, including ischaemic stroke and myocardial infarction.

Venous thromboses are a well-documented complication of malignancy in general and specifically have a notably high incidence in pancreatic malignancy [2]. However, the prevalence and incidence of arterial disease, including ischaemic stroke and myocardial infarction, are less well documented. Existing research suggests that the co-occurrence of arterial thromboembolism in patients with underlying malignancy is associated with an increased risk of mortality compared to those without arterial events [3].

While stroke remains a leading cause of death worldwide, its management in the context of malignancy-associated hypercoagulability is complex. Research has demonstrated that stroke patients with Trousseau syndrome have a worse prognosis compared to those with other causes of cerebral infarct [4]. Large cohort data also show a 91% higher 12-month mortality (HR 1.91, 95% CI 1.50-2.43) and a 68% higher risk of cerebrovascular recurrence (HR 1.68, 95% CI 1.22-2.31) in patients with active cancer compared with non-cancer patients [5]. This underscores the need for improved understanding of cancer-associated stroke to optimise outcomes.

The pathophysiology of cancer-related hypercoagulability is multifactorial. Tumour cells may overexpress tissue factor and release tissue factor-bearing microparticles, mucin, and inflammatory cytokines, which activate endothelial cells, initiate the coagulation cascade, and enhance platelet aggregation. These processes promote thrombin generation and fibrin deposition, creating a systemic prothrombotic state. Additional mechanisms include non-bacterial thrombotic endocarditis (NBTE), direct intravascular coagulation, and tumour-induced platelet activation [6].

We present a complex case involving a patient with mixed arterial ischaemic disease and venous thromboembolic disease that unearthed a diagnosis of metastatic pancreatic cancer, a case of Trousseau syndrome.

## Case presentation

We report a case of a 70-year-old male with a past medical history of hypertension and type 2 diabetes mellitus. He was functionally independent, a non-smoker, and consumed minimal alcohol. He presented to the Emergency Department with expressive dysphasia and a right-sided facial weakness.

On first presentation, he reported a sudden onset of word-finding difficulty. On examination, his speech was non-fluent, with features of expressive and receptive dysphasia and a mild right-sided facial droop. Limb power and sensation were preserved. His observations were within normal limits, and cardiovascular, respiratory, and abdominal examinations were unremarkable.

**Figure 1:**
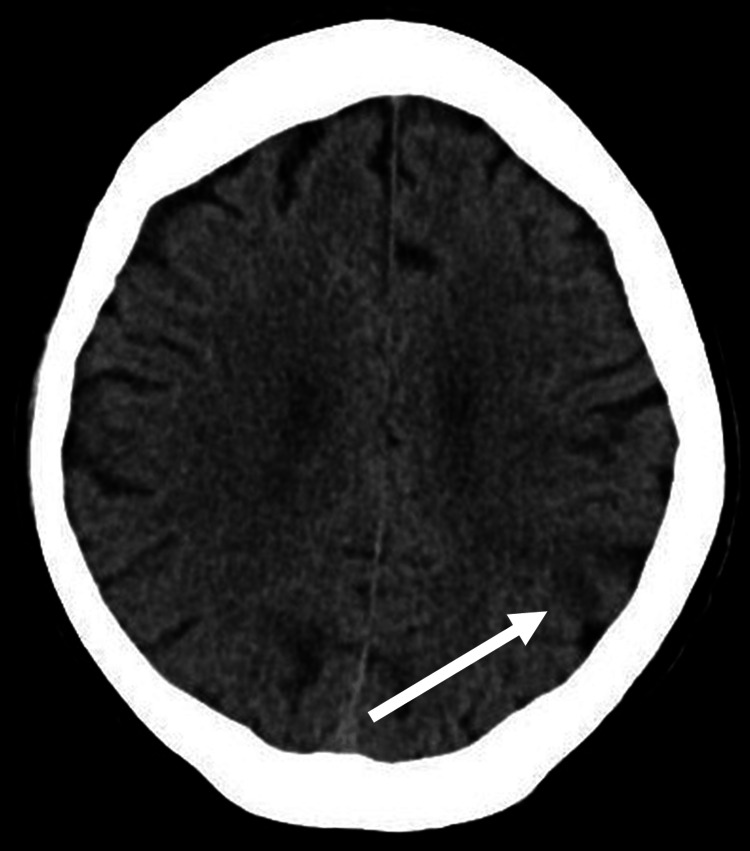
CT head showing acute infarct of the left parietal lobe. The arrow indicates the area of hypodensity indicative of an acute stroke. Axial non-contrast CT image showing an area of hypodensity in the left parietal lobe, consistent with an acute ischaemic infarct in the middle cerebral artery territory. (Image obtained from the hospital PACS system with patient consent.) The arrow indicates the area of hypodensity indicative of an acute stroke. PACS: Primary Agricultural Credit Societies

Blood tests on review revealed white cell count of 15.3 × 10^9/L (normal range 4.0-11.0 × 10^9/L), C-reactive protein of 38 mg/L (Normal range <10 mg/L), but otherwise his biochemistry was unremarkable. The electrocardiogram showed a normal sinus rhythm. A non-contrast computed tomography (CT) scan of the brain demonstrated an acute infarct in the left parietal lobe (Figure 1). He was diagnosed with a left middle cerebral artery ischaemic stroke.

A CT angiogram of the intracranial vessels was performed as part of the stroke work-up. Although the primary purpose was to assess the intracranial vasculature, the scan also visualised the upper lung fields and incidentally revealed small pulmonary nodules.

Following multidisciplinary stroke assessment, he was then discharged on high-dose atorvastatin and aspirin for two weeks, with an urgent outpatient imaging to further investigate for an underlying malignancy.

**Figure 2:**
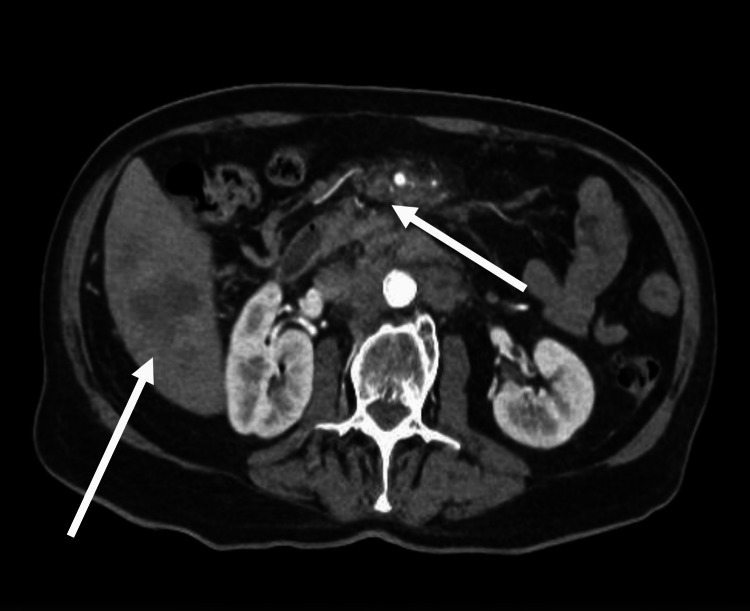
Computed Tomography of the abdomen and pelvis showing primary pancreatic malignancy with liver metastasis. Axial contrast-enhanced CT image showing a pancreatic mass with associated hepatic metastases. (Image obtained from the hospital PACS system with patient consent.) Arrows indicate liver metastases and pancreatic malignancy.

Subsequent contrast-enhanced CT of the thorax, abdomen, and pelvis demonstrated a primary pancreatic malignancy with liver (Figure 2) and lung metastases, as well as small segmental pulmonary emboli in both lower lobes. The markedly raised tumour marker CA19-9 and radiological evidence of metastatic disease supported the diagnosis of advanced pancreatic adenocarcinoma.

Therapeutic anticoagulation was initiated with apixaban at a dose of 10 mg twice daily for seven days, followed by 5 mg twice daily, in line with standard treatment for acute pulmonary embolism in a clinically stable patient. He had normal renal function and no contraindications to direct oral anticoagulant (DOAC) therapy. The patient was referred to the local upper gastrointestinal multidisciplinary team for further evaluation and management of metastatic pancreatic cancer.

Second admission: Recurrent bilateral extensive cerebral and cerebellar strokes

Several weeks later, while taking apixaban 5 mg twice daily, the patient was re-admitted after being found collapsed at home. On arrival, he had a right homonymous hemianopia and mixed expressive and receptive dysphasia.

On examination, auscultation revealed coarse crackles over the right lung field, with normal heart sounds. Abdominal examination demonstrated right upper quadrant tenderness. Neurological assessment was limited by poor cooperation, but limb power was preserved (Medical Research Council grade 5/5 throughout) and sensation was intact. He was disoriented in time, person, and place and was unable to follow commands. A right homonymous hemianopia and dysarthria were noted.

Blood tests during this admission showed markedly elevated inflammatory, cholestatic markers and CA19-9 (Table 1), indicating a very high tumour burden. The alkaline phosphatase (ALP) was 1,268 U/L (reference range 30-130 U/L), supporting significant hepatic involvement from metastatic disease. The white cell count was 21.3 × 10⁹/L and CRP were 179 mg/L, consistent with systemic inflammation and possible biliary sepsis.

Electrocardiography demonstrated a normal sinus rhythm.

**Table 1:** Blood test results from the second admission, showing raised inflammatory markers, tumour marker and ALP. ALP: alkaline phosphatase

Test	Result	Reference range
Ca19-9	74,335 kU/L	0-37 kU/L
White cell count	21.3 × 10⁹/L	4.0–11.0 × 10⁹/L
Alkaline phosphatase	1,268 U/L	30–130 U/L
C-reactive protein	179 mg/L	<10 mg/L

The initial differential diagnosis included biliary infection and recurrent acute ischaemic stroke. Apixaban was temporarily withheld while intracranial haemorrhage was excluded, and the patient was commenced on empirical antibiotics.

**Figure 3:**
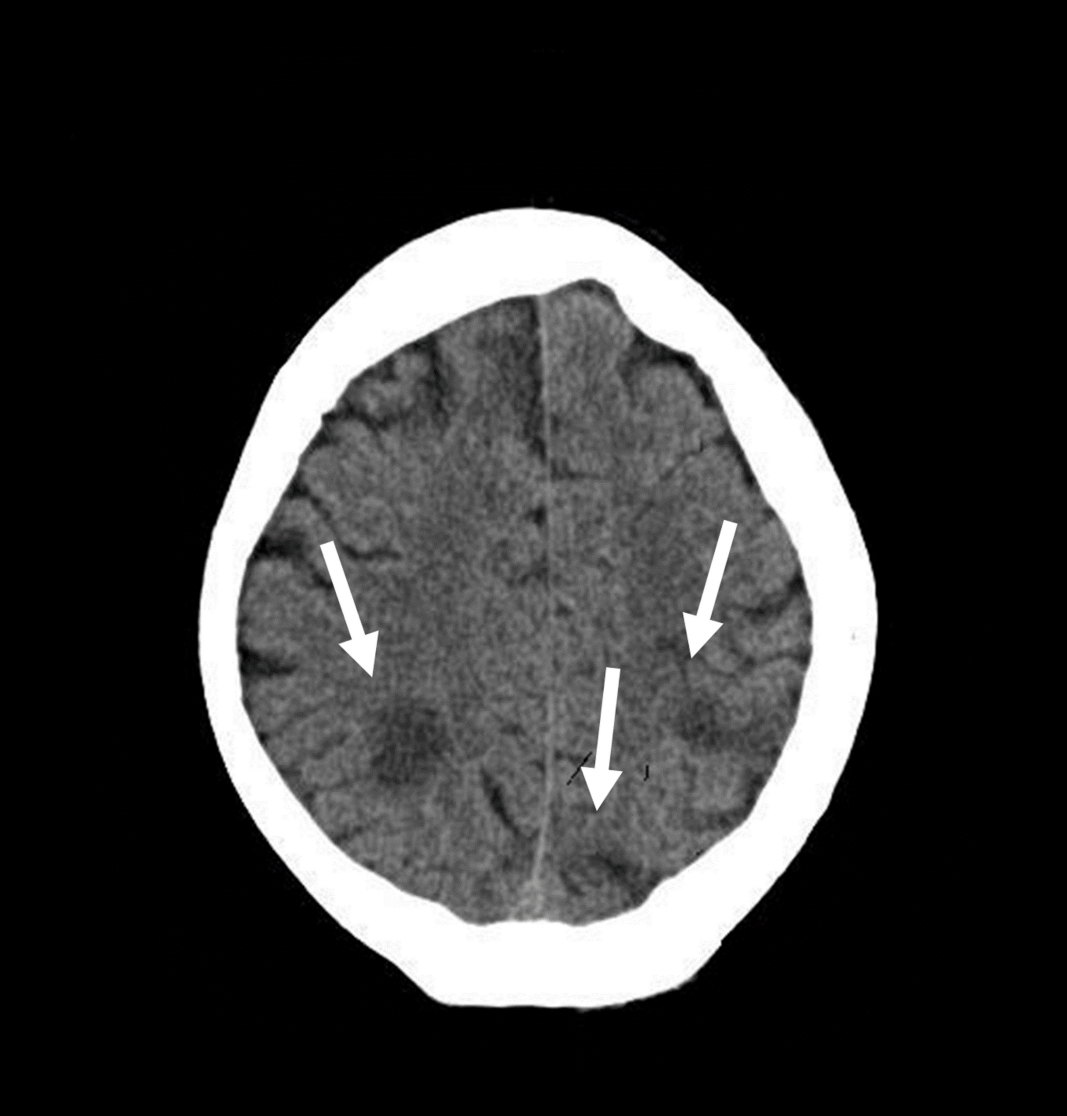
CT head with contrast showing new bilateral parietal, left parieto-occipital and right frontal lobes infarcts. Axial non-contrast CT image demonstrating new areas of hypodensity involving the bilateral parietal, left parieto-occipital, and right frontal lobes, consistent with recent multifocal ischaemic infarcts. (Image obtained from the hospital PACS system with patient consent.) Arrows indicate multiple areas of hypodensity, indicating acute infarcts.

A non-contrast CT scan of the brain was performed to exclude haemorrhagic transformation or spontaneous intracranial bleeding. CT imaging demonstrated new bilateral parietal, left parieto-occipital, and right frontal lobe hypodense changes, consistent with multifocal ischaemic infarcts (Figure 3). 

**Figure 4:**
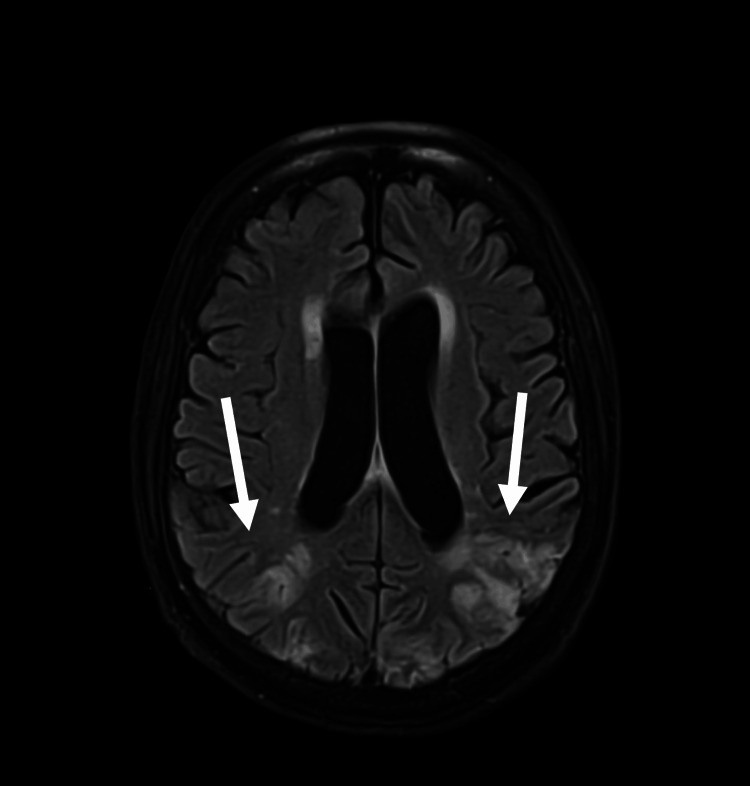
MRI with contrast showing multiple zones of acute ischaemia in the cerebral and cerebellar hemispheres bilaterally. Axial MRI brain with contrast showing multifocal acute ischaemic changes involving both cerebral and cerebellar hemispheres, most pronounced in the posterior watershed territories. (Image obtained from the hospital PACS system with patient consent.) Arrows indicate areas of hyperdensity, confirming multiple acute ischaemic infarcts.

Magnetic resonance imaging (MRI) of the brain with contrast was performed to evaluate for intracerebral metastatic disease and further evidence of acute ischaemic stroke. The MRI demonstrated multiple zones of acute ischaemia involving both the cerebral and cerebellar hemispheres, most prominently in the posterior watershed territories. The appearances were suggestive of a large embolic event (Figure 4).

The patient was subsequently referred to the stroke team and admitted to the high-acuity stroke unit, where further examination confirmed a right homonymous hemianopia and dysarthria with poor limb coordination.

Transthoracic echocardiography (TTE) was performed to assess for a potential cardiac source of emboli. This demonstrated mildly impaired left ventricular systolic function with an ejection fraction of 45% but no evidence of intracardiac thrombus or valvular vegetations. However, NBTE could not be definitively excluded, as TTE has limited sensitivity for detecting small valvular lesions. Transoesophageal echocardiography (TOE), which is more sensitive for NBTE, was not performed given the patient’s advanced malignancy, frailty, and overall poor prognosis.

## Discussion

Trousseau syndrome, first described by Armand Trousseau in the 1860s, refers to a malignancy-associated hypercoagulable state that can manifest as migratory thrombophlebitis, venous thromboembolism, and arterial thrombosis [1].

Pancreatic cancer is strongly associated with this syndrome, and other mucin-producing cancers (gastric and lung) are also implicated [2,6,7,8]. Trousseau’s clinical spectrum may represent the first clinical sign of an occult malignancy and should prompt clinicians to investigate for an underlying neoplasm.

The pathophysiology of malignancy-related thrombosis is multifactorial and complex. Several mechanisms have been proposed, including NBTE, direct intravascular coagulation, and systemic platelet activation driven by tumour-derived procoagulant factors.

Pancreatic adenocarcinoma is highly thrombogenic due to overexpression of tissue factor and the release of tissue factor-bearing microparticles, mucin, and inflammatory cytokines. These factors activate endothelial cells, initiate the coagulation cascade and platelet aggregation, and promote thrombin generation and fibrin deposition, collectively creating a systemic hypercoagulable state.

Recent evidence suggests that tumour-derived extracellular vesicles and neutrophil extracellular traps (NETs) further amplify this prothrombotic state. These structures enhance platelet aggregation, activate the coagulation cascade, and promote endothelial dysfunction, thereby intensifying systemic hypercoagulability beyond the effects of tissue factor and mucin alone [9,10,11].

This case demonstrated the classic presentation of Trousseau syndrome, in which an initially cryptogenic stroke led to the discovery of metastatic pancreatic cancer and pulmonary emboli, followed by recurrent ischaemic strokes. Despite treatment with apixaban, our patient developed further multifocal bilateral ischaemic strokes, reflecting the intensity of the tumour-driven prothrombotic process. The distribution of infarcts involving multiple vascular territories, including the posterior watershed zones, is typical of embolic showers of microthrombi formation secondary to a hypercoagulable state rather than a single embolic source.

The risk of venous thromboembolism (VTE) is a frequent complication in patients with cancer, with approximately a 15-fold higher risk compared with those without cancer [6,11]. Patients with active cancer who develop VTE have a markedly increased risk of recurrent thrombosis (15-20% within six months), even while on anticoagulation, due to continuous tumour-driven release of procoagulant factors. In addition, cancer-associated VTE carries a higher mortality risk than non-cancer VTE.

While direct oral anticoagulants (DOACs), such as apixaban, are widely used for the treatment of VTE, studies have shown that DOACs may be less effective in mucin-producing adenocarcinomas [12]. Current guidelines recommend low-molecular-weight heparin (LMWH) as the first-line treatment for cancer-associated thrombosis (CAT), as demonstrated in the CLOT trial, which showed a lower recurrence rate with LMWH compared to vitamin K antagonists. LMWH also exhibits anti-tumour and anti-angiogenic effects, potentially offering broader benefits.

In our patient, the recurrence of strokes and pulmonary emboli despite anticoagulation complicated his treatment plan, rendering him unfit for chemotherapy and highlighting the aggressive, refractory nature of Trousseau syndrome. This case underscores the importance of recognising recurrent thrombotic events as a potential presentation of an underlying occult malignancy, particularly when affecting multiple vascular territories.

## Conclusions

This case illustrates a severe manifestation of Trousseau syndrome, characterised by recurrent ischaemic strokes and pulmonary emboli in the setting of metastatic pancreatic cancer, despite therapeutic anticoagulation with apixaban. The patient’s clinical course demonstrates the intensity of the tumour-driven hypercoagulable state and the challenges in preventing further thromboembolic events.

Clinicians should maintain a high index of suspicion for occult malignancy in patients presenting with unexplained or recurrent venous and arterial thromboembolic events, particularly when multiple vascular territories are involved. Early recognition and appropriate investigation can facilitate timely cancer diagnosis and inform multidisciplinary management, although treatment options may remain limited in the context of advanced disease and aggressive hypercoagulability.
